# Primitive neuro-ectodermal tumor of the lung in an adult

**DOI:** 10.4103/0970-2113.53233

**Published:** 2009

**Authors:** G. S. Gaude, P. R. Malur, R. Kangale, S. Anurshetru

**Affiliations:** *Department of Pulmonary Medicine, J. N. Medical College, Belgaum, Karnataka, India*; 1 *Department of Pathology, J. N. Medical College, Belgaum, Karnataka, India*; 2 *Department of Cardiothoracic Surgery, J. N. Medical College, Belgaum, Karnataka, India*

**Keywords:** Primitive neuroectodermal tumor, mediastinum, thoracotomy

## Abstract

A rare case of a thoracic primitive neuro-ectodermal tumor in an adult is presented here. In this case, wide excision surgical excision followed by chemotherapy and radiotherapy were delivered. But due to the rapid aggressive progression of the tumor, which is the characteristic of disease, the patient died within four months after the diagnosis.

## INTRODUCTION

Primitive neuro-ectodermal tumors (PNET), described originally by Askin and colleagues,[1] are infrequent thoracic tumors found in infants and children.[2] The histogenesis of this tumor remains uncertain and is suspected to arise from the intercoastal nerves.[3] Frequently, this entity appears as a chest wall mass, with rapid growth that may involve the pleura.[4] It tends to occur in young females, it is seen exclusively in one hemithorax, and rib destruction is a frequent complication. The condition is exceedingly rare in adults. We herein report a case of PNET entity in an adult having a rapid progression of the disease.

## CASE REPORT

**Figure 1:**
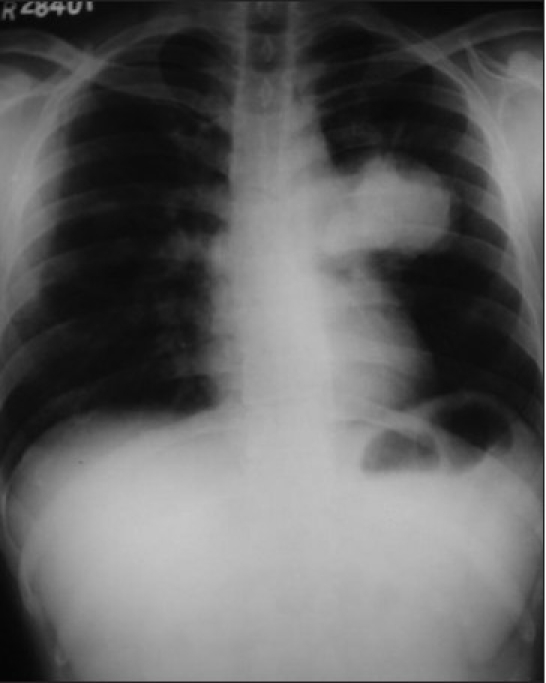
Chest radiograph showing large well defined mass near the left hilum

**Figure 2:**
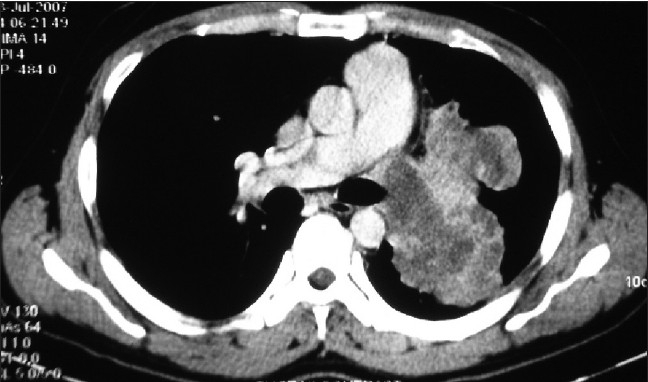
CT scan of the thorax showing large well defined lobulated, nonhomogenous mass extending up to the pleural surface

**Figure 3:**
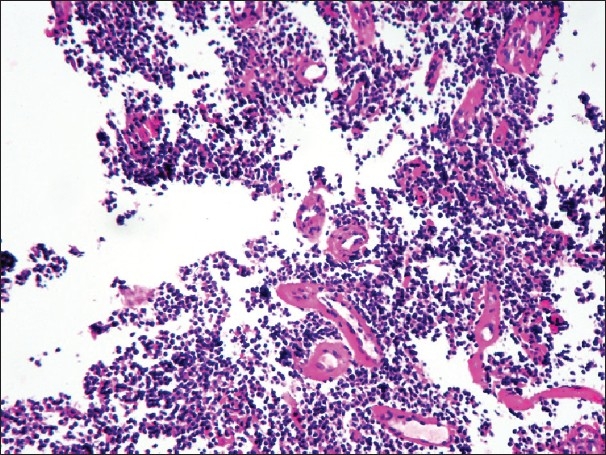
Biopsy specimen of the PNET tumor showing tumor cells arranged in nests. These cells are perivascular in arrangement. Cells are PAS positive. (H and E, ×40)

A 28-year old male, presented with chest pain and mild dyspnea, of 20 days duration. He was a farmer and nonsmoker with no history of exposure to any occupational or inorganic dusts. The chest pain was retrosternal and pricking in character with no radiation and not related to the meals. Examination revealed averagely built person and no clubbing and lymphadenopathy. Respiratory system evaluation revealed signs of mass in the left hemithorax. Chest radiograph revealed a large mass near the left hilum [Figure 1]. Routine blood investigations were within normal limits. Computed tomography (CT) of the thorax revealed a large well defined, nonhomogeneous mediastinal mass, in the left hemithorax near the hilum. The mass was extending up to the pleural surface [Figure 2]. There was an involvement of the mediastinal lymph nodes. Fiberoptic bronchoscopy revealed extrinsic compression of left upper lobe bronchus with no intra bronchial extension. Bronchial washings, bronchial brush biopsy, and transbronchial needle aspiration biopsy of the mass ware inconclusive. Transthoracic fine needle aspiration biopsy of the mass revealed small round tumor cells with thin strands of fibrous connective tissue. As the diagnosis was inconclusive, the patient was taken up for thoracotomy with excision of the tumor. Intraoperatively it was observed that there was a large mass in the mediastinum with invasion of the great vessels and the mediastinal lymph nodes. Hence, tumor debulking was done to the extent possible. Complete excision of the tumor could not be done as it was infiltrating the great vessels and the pericardium. The tumor had extended up to the chest wall. Histologically, the specimen revealed round to oval tumor cells with scanty cytoplasm and hyper chromatic nuclei. These neoplastic cells also showed rosette formation [Figure 3]. Immunohistochemistry of the specimen proved the diagnosis of PNET with positivity of the synaptophysins and chromogramin. As the diagnosis of primitive neuroectodermal tumor was established, and wide surgical excision of the tumor was already been done, the patient was referred for radiotherapy and chemotherapy. The patient received radiotherapy with 4,000 Rads and chemotherapy with cisplatinum, bleomycin, and doxorubicin. The patient continued to be symptomatic, and his condition worsened over a period of next three months and he died subsequently four months after the diagnosis.

## DISCUSSION

PNET are rare tumors occurring in the posterior sulcus or chest wall of adolescent or young adult patients. They are believed to develop from the peripheral nerves such as an intecoastal nerves. They are highly aggressive neoplasms of the thorax with a median survival of eight months. They are typically painful, invasive thoracic tumors that may develop on and invade chest wall, lung, or mediastinum.[5] They are generally soft and fleshy with areas of hemorrhage and necrosis. Although the tumors were classified by Askin *et al.*[1] with thoracic autonomic neurogenic tumors, the PNET cells do not produce biologically active substances detectable in the blood or urine. A similar chromosomal translocation occurring in both Ewing's sarcoma and PNET lesions suggest that these tumors are closely related. Typically, Askin's tumor develops as a solitary mass or multiple masses in the thoracic area. Pain is the only or main symptom in 60% of the cases. In the thoracic area, these tumors are invasive and prone to destroying bone, invading the retroperitoneal space, and spreading to lymph nodes, adrenals, and liver. Once resected, they recur with extremely high frequency. The most common recurrence sites are the skeleton, the sympathetic chain, and the original site. Image diagnosis is based on chest radiographs, CT scan and MRI, the latter having the greatest sensitivity. Fine needle aspiration biopsy identifies the presence of small cells with neurosecretory granules, microtubules, and cytoplasmic extensions.[6] Results of 123I-Tyr-3-Octeotride scintigraphy are positive for most of the tumors and metastasis.[7] Characteristics PENT pathologic findings include compact nests of small cells, Homer-Wright pseudo rosettes with an acidophilic core of neurofibrillar characteristics, and uptake of neuron specific enolase stain.[8]

The treatment for PENT must consist, wherever possible, radical resection supplemented with radiotherapy (3,500 to 5,500 Rads) and aggressive chemotherapy (cyclophosphamide, vincristine, doxorubicin, cisplatinum, methotrexate, bleomycin, and dimethyltriazenyl imidazole carboseamide). The average life expectancy is eight months, although occasional long-term survival can be as long as 96 years.[6]

The present case describes one of the few reported cases of PNET in an adult. It is unusual for the age of presentation and a short duration of unique symptom i.e., thoracic pain, which had started only few days before hospital admission. Plain chest radiograph and CT scan were diagnostic for pulmonary tumor and FNAC was diagnostic for a small cell tumor with a high suspicion of malignancy. Open thoracotomy with excision of the removable tumor proved the diagnosis of PNET. The tumor had invaded the great vessels in the thorax and the mediastinal lymph nodes due to the advanced nature of the tumor.

Primitive neuroectodermal tumor lesions are aggressive and are usually lethal; they should be considered in the differential diagnosis of thoracic tumors regardless of the age of the patient. Once the diagnosis of the PNET has been made, early wide excision of the tumor, along with multimodality chemotherapy and radiotherapy, should be undertaken to offer any hope of a long-term cure.
